# Pt Single Atom Co‐Catalysts on Thin, Defined Anatase Layers: Critical Factors for Photocatalytic Hydrogen Generation

**DOI:** 10.1002/smll.202507662

**Published:** 2025-09-12

**Authors:** Hyesung Kim, Nikita Denisov, Yue Wang, Patrik Schmuki

**Affiliations:** ^1^ Department of Materials Science WW4‐LKO Friedrich‐Alexander‐University of Erlangen‐Nuremberg Martensstrasse 7 91058 Erlangen Germany; ^2^ Regional Centre of Advanced Technologies and Materials Šlechtitelů 27 Olomouc 78371 Czech Republic

**Keywords:** hydrogen generation, Pt single atoms, reactive DC magnetron sputtering, thin film deposition photocatalysis, TiO_2_

## Abstract

Single‐atom catalysts (SACs) have emerged as promising co‐catalysts in photocatalytic hydrogen generation. However, key challenges remain in this field, particularly in reliably controlling their surface density, dispersion, and integration into semiconductor systems. This study systematically investigates platinum single atoms deposited on anatase TiO_2_ thin films, grown on fluorine‐doped tin oxide with defined thickness and crystallographic structure, by direct‐current sputter‐deposition of titania. Comprehensive parameter screening shows that variations in Pt precursor concentration, pre‐annealing temperature, and TiO_2_ layer thickness significantly influence the photocatalytic performance. Crucially, a Langmuir‐type adsorption behavior for Pt single atoms is demonstrated and extract an optimal surface density of ≈4 × 10^5^ SAs µm^−2^ (around 0.26 at.% Pt) – higher loading does not further enhance photocatalytic activity. For optimized co‐catalyst loading, variations in TiO_2_ thickness and structure remain the primary factors influencing photocatalytic performance through charge transport, and light absorption as key parameters. This optimal loading is confined to the surface of the TiO_2_ film and is sufficient to fully utilize the photogenerated electron flux under ultravoilet excitation. This work introduces the concept of a critical co‐catalyst density that separates the transition from co‐catalyst‐limited to absorber‐limited behavior in SAC‐based photocatalysis, thereby contributing to a further rational design of photocatalytic systems.

## Graphical abstract

Optimized Pt single atoms are deposited on sputter‐grown TiO_2_ films with well‐controlled crystallinity and thickness. A saturation in hydrogen evolution is observed at ∼0.26 at.% Pt, indicating a critical co‐catalyst threshold. Beyond this, photocatalytic performance depends primarily on absorber properties, highlighting the importance of balancing catalyst loading with optical design to unlock the full potential of single‐atom‐driven solar hydrogen generation.

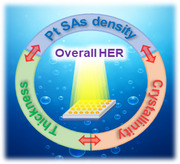

## 1 Introduction

Single atom catalysis (SAC), over the recent years, has become a highly investigated topic in heterogeneous catalysis,^[^ ^1^, ^2^, ^3^, ^4^, ^5^, ^6^, ^7^, ^8^ ^]^ and supported single atoms (SAs) have been explored for a wide range of “classic” noble‐metal‐catalyzed reactions.^[^ ^8^, ^9^, ^10^, ^11^, ^12^, ^13^, ^14^, ^15^, ^16^, ^17^ ^]^ This is on the one hand because of economic reasons, i.e., the goal to minimize the use of noble metals, but also due to entirely novel reaction pathways (unique selectivity) that can become available using SAs.^[^ ^18^, ^19^, ^20^, ^21^ ^]^ Recently, SAs have also attracted significant interest in photocatalysis, where they are intensively explored as co‐catalysts in photocatalytic or photoelectrochemical reactions, such as pollution remediation or the generation of H_2_ from aqueous solutions.^[^ ^3^, ^8^, ^22^, ^23^, ^24^, ^25^, ^26^, ^27^ ^]^ In the latter process, a semiconductor is illuminated with super bandgap (solar) light (*hv* > E_g_), and the generated charge carriers after diffusion to the surface initiate oxidation and reduction reactions (such as the reduction of H_2_O or H^+^ to H_2_ and the corresponding oxidation of water or a sacrificial agent). However, on many oxide surfaces, the reaction of photogenerated electrons with H_2_O or H^+^ is kinetically hindered, and H_2_ production remains sluggish. To overcome these issues, semiconductors are frequently decorated with so‐called co‐catalysts that are typically noble metals such as Pt, Pd, Rh, etc., that are usually deposited in the form of nanoparticles on the semiconductor surface.^[^ ^28^, ^29^, ^30^ ^]^ For TiO_2_, the most widely used semiconductor in photocatalysis, the use of Pt nanoparticles as a H_2_ evolution co‐catalyst is well established.^[^ ^31^, ^32^, ^33^, ^34^, ^35^ ^]^ To deposit Pt nanoparticles on titania, a range of solution‐based or light‐induced reduction techniques has been used.^[^ ^36^, ^37^, ^38^, ^39^, ^40^ ^]^ For the deposition of SAs onto titania substrates, a variety of chemical and physical deposition strategies have been developed.^[^ ^41^, ^42^, ^43^ ^]^ Particularly straightforward are techniques based on immersion of TiO_2_ into a Pt‐precursor solution that either rely on strong electrostatic adsorption^[^ ^8^, ^44^, ^45^, ^46^, ^47^ ^]^ or alternatively, SAs can be attached to TiO_2_ surfaces by a reactive deposition approach.^[^ ^8^, ^48^, ^49^, ^50^ ^]^ The latter relies on the reaction of H_2_PtCl_6_ with defects either naturally present on titania or fabricated by suitable defect engineering approaches.^[^ ^22^, ^23^, ^48^, ^49^, ^50^ ^]^ SAs deposited using a reactive approach can achieve a remarkable photocatalytic H_2_ evolution performance using only comparably small amounts of Pt,^[^ ^22^, ^48^, ^49^, ^50^, ^51^, ^52^ ^]^ Ir,^[^ ^53^ ^]^ or Pd.^[^ ^23^ ^]^ Nevertheless, the vast majority of investigations of SA co‐catalysts use complex substrates such as powders or nanostructures that complicate a straightforward study of the influence of substrate and SA parameters on the system performance.

In the present study, we use a geometrically well‐defined flat semiconductor thin‐layer model to systematically investigate SA and semiconductor contributions in view of photocatalytic H_2_ generation. For this, compact TiO_2_ layers are deposited onto fluorine‐doped tin oxide (FTO) glass substrates via direct current magnetron sputtering (DC‐MS)—this allows for precise absorber properties such as layer thickness and crystallographic structure.^[^ ^54^ ^]^ These layers are subsequently decorated with single‐atom (SA) Pt species through “reactive deposition” from dilute H_2_PtCl_6_ solutions.^[^ ^8^, ^48^, ^49^, ^50^ ^]^ Systematic variation of parameters such as layer thickness, annealing conditions, Pt loading, and excitation wavelength allows for a comprehensive assessment of their effects on H_2_ generation performance. Our findings allow us to extract and optimize the Pt/TiO_2_ system for controllable photocatalytic H_2_ generation and minimize noble metal usage.

## 2 Results and Discussion

**Figure 1:**
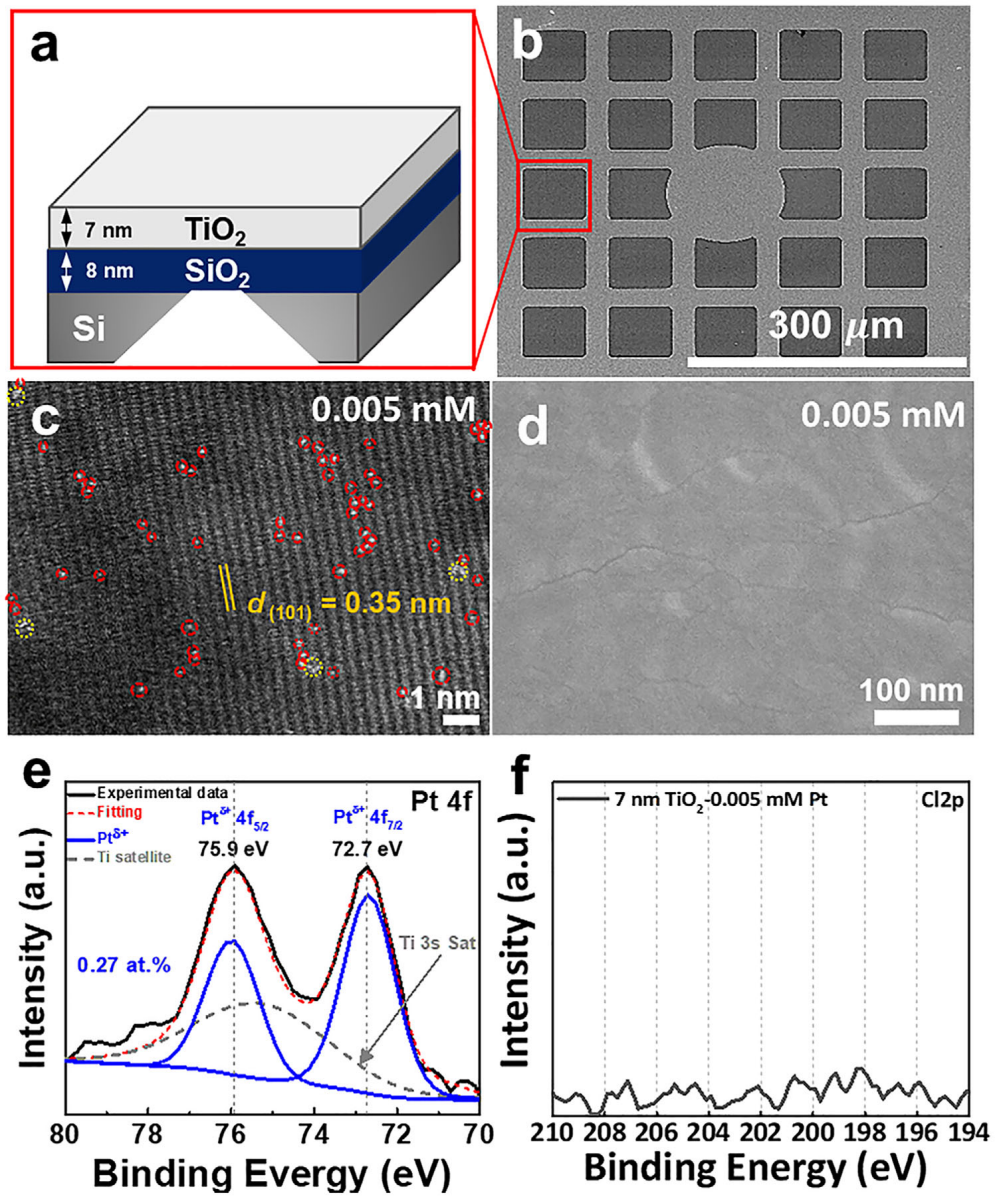
a) Schematic drawing of the sputtered TiO_2_ layer on TEM grid, b) SEM image of the 7‐nm TiO_2_ deposited onto the 8‐nm SiO_2_ TEM window, c) HAADF‐TEM image of sputtered 7‐nm‐TiO_2_ layer decorated with 0.005 mm H_2_PtCl_6_ precursor solution on TEM grid, d) SEM image of 7‐nm‐sputtered TiO_2_ layer on SiO_2_/Si wafer with 0.005 mm Pt solution and e) XPS spectra of Pt 4f and f) Cl 2p for 0.005 mm Pt decorated TiO2 layer on SiO_2_/Si wafer.

In our experiments, first thin film TiO_2_ layers were deposited on FTO substrates by DC‐MS, then typically annealed to anatase, as described in the experimental section. The layers then were decorated with Pt SAs by reactive deposition (immersion in dilute H_2_PtCl_6_ solutions, as described previously^[^ ^48^, ^49^, ^51^ ^]^). For an initial screening, TiO_2_ layers were sputtered to a thickness of ≈ 200 nm on FTO or to a thickness of 7 nm on photolithographically produced transmission electron microscopy (TEM) grids, as illustrated in **Figure** **1**a. The latter were then used to detect Pt SAs by high‐angle annular dark‐field scanning transmission electron microscopy (HAADF‐STEM). Figure 1b shows a scanning electron microscopy (SEM) image of such a TEM grid, and Figure 1c shows a HAADF‐TEM image of a 7 nm TiO_2_ layer where reactive Pt‐deposition was carried out by exposure to 0.005 mm H_2_PtCl_6_ solution for 1 h. Deposition of Pt SAs (red circles) and a few 2D‐rafts (yellow circles) can be seen on the substrate–the substrate shows the characteristic (101) anatase lattice spacing of 0.35 nm.^[^ ^49^ ^]^ Although the distribution of Pt atoms appears locally with some degree of inhomogeneity at the nanoscale, statistical analysis of multiple HAADF‐STEM images confirms a consistent average SA density of ≈4.1 × 10^5^ µm^−2^ across different regions. Figure 1c thus primarily serves to demonstrate that, under these deposition conditions, the majority of Pt species are present as atomically dispersed single atoms on the TiO_2_ surface. This observation highlights the effectiveness of the reactive deposition approach in achieving well‐dispersed Pt at low concentrations. Importantly, in a high‐resolution SEM image of the Pt decorated titania surface (Figure 1d), no distinct particles can be seen—this shows that over the entire surface area, no agglomeration of SAs to nanoparticles in the nm‐range has taken place (Pt agglomeration can be detected by field emission‐scanning electron microscopy (FE‐SEM) if the Pt particles are larger than ≈0.8 nm). X‐ray photoelectron spectra (XPS) of the surface show for the Pt 4f region (Figure 1e) the presence of Pt (0.27 at.%), evident with a doublet of Pt 4f_7/2_ and 4f_5/2_ at peak positions of 72.7 and 75.9 eV, respectively. This position is typical of Pt SAs coordinated with an oxide surface^[^ ^22^, ^55^ ^]^ where the SA carries a formal charge on the Pt^δ+^ with δ ≈ 2. Please note, this peak position is significantly different from metallic Pt^0^ (in nanoparticles), where the doublet Pt 4f_7/2_ and 4f_5/2_ peaks are shifted to ≈71.0 and ≈74.5 eV (for example, see Figure S1b,c, Supporting Information).^[^ ^56^, ^57^, ^58^, ^59^ ^]^ Importantly, for this sample, the XPS spectrum in the Cl 2p region (Figure 1f) shows the absence of Cl, i.e., no remaining chlorine coordination to Pt can be detected, reflecting complete reaction of the H_2_PtCl_6_ during dissociation in water and reaction with the titania surface.^[^ ^8^, ^48^, ^55^ ^]^ These findings show that the combination of SEM (no identifiable particles) with XPS (characteristic peak position) can be used to provide evidence for the presence and the amount (loading) of SAs on flat surfaces (as also outlined in previous work.^[^ ^48^, ^49^, ^51^, ^55^ ^]^ The advantage of this SA identification approach is that it can be applied to any flat surface, and in particular, it does not require TEM‐transparent substrates. In our case, this is of high value for photocatalytic investigations, where we need thicker layers to provide a sufficient light absorption depth—and accordingly on theses layers, we characterized the Pt speciation and loading using SEM and XPS.^[^ ^48^, ^55^, ^57^ ^]^

**Figure 2:**
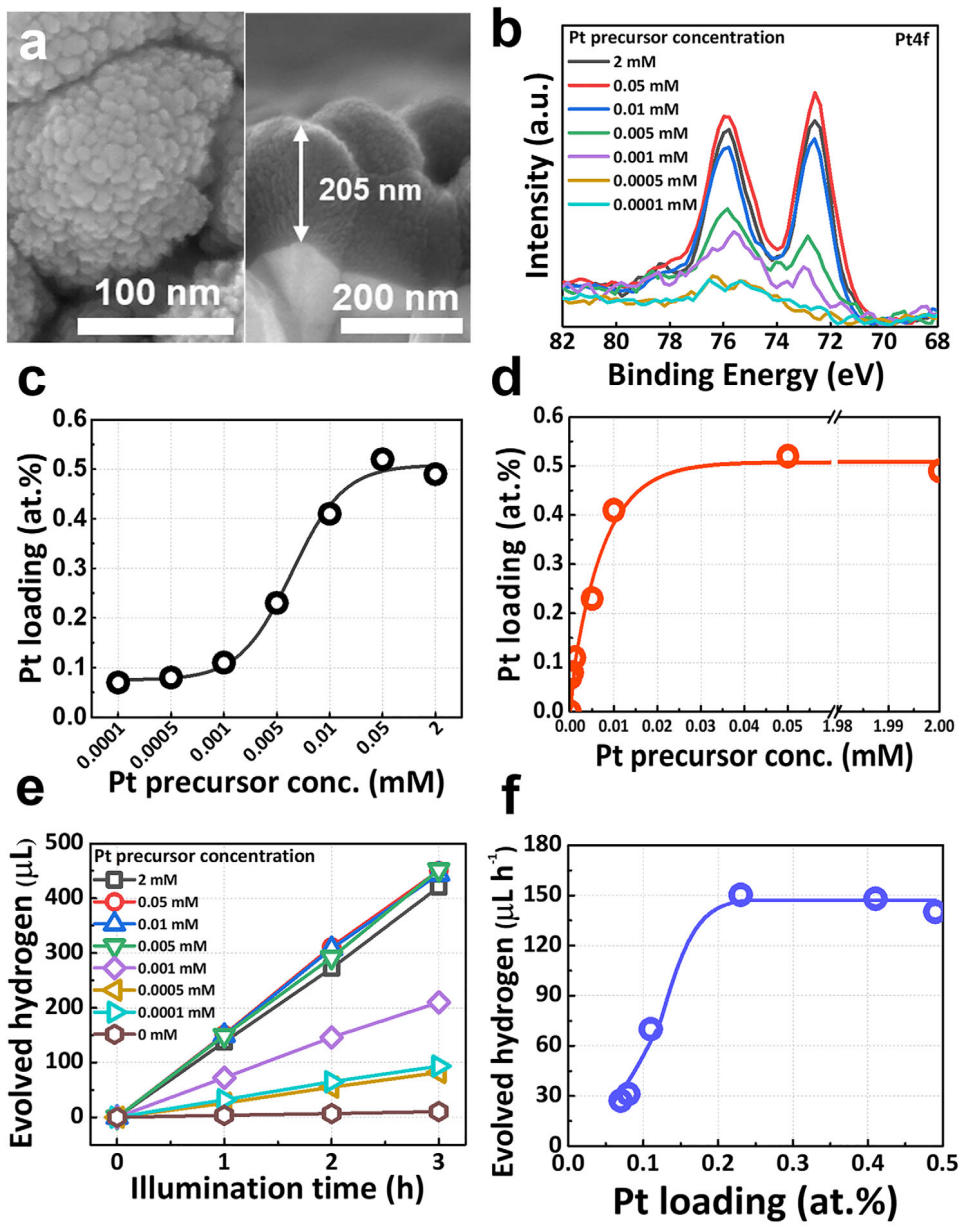
a) Top and cross‐sectional SEM images of 200‐nm‐thick TiO2 layer sputtered on FTO substrate, b) XPS spectra of Pt 4f on titania surface deposited in various concentrations of H_2_PtCl_6_ precursor solution, c) Pt precursor concentration versus Pt surface loading on TiO_2_, d) Langmuir isotherm for Pt loading, e) photocatalytic hydrogen production for 3 h, and f) H_2_ production rate as a function of Pt surface loading on titania.

We started parameter screening using 200‐nm‐thick TiO_2_ layers on FTO substrate as in the SEM images (**Figure** **2**a) by varying the Pt precursor (H_2_PtCl_6_) concentration in the range of 0.0001–2 mm. From SEM, for none of the titania layers, agglomerated Pt nanoparticle could be detected (Figure S2, Supporting Information), and across all samples, the XPS analysis (Figure 2b) revealed clear SA characteristics with Pt 4f_7/2_ and 4f_5/2_ signals at ≈72.6 and ≈75.9 eV (i.e., Pt‐SAs) without any residual Cl peaks (Figure S3, Supporting Information). The Pt SA loadings, extracted from XPS, on the titania surface are given in **Table** **1**, and the loading as a function of the precursor concentration is shown in Figure 2c. Evidently, at low solution concentrations (from 0.0001 to 0.01 mm), the Pt loading increases steadily until it reaches saturation at ≈0.4 at.% for the 0.01 mm solution. For higher concentrations than 0.01 mm, no higher Pt SA loading can be achieved. This saturation behavior is well in line with a Langmuir‐type adsorption characteristic (Figure 2d) and suggests that there is a finite amount of surface docking sites on the substrate layer where Pt SA deposition takes place. In previous work, we described the attachment process as a galvanic displacement reaction of surface Ti^3+^ sites on TiO_2_ with Pt^4+^, resulting in nominally Pt^2+^‐attached SAs.^[^ ^48^ ^]^ Thus, one may stipulate that once these docking sites are used up, there is saturation in attachment, and at this point, all accessible adsorption active sites on the TiO_2_ surface are occupied by Pt atoms, preventing further adsorption even for very high precursor concentrations.

**Table 1:** Deposited Pt content (by XPS) on 200‐nm TiO_2_ layers for different Pt precursor concentrations and corresponding H_2_ production rates.

Pt precursor concentration [mm]	Pt content [at. %]	H_2_ production rate [µL h^−1^]
2	0.49	140.2
0.05	0.52	150.1
0.01	0.41	148.1
0.005	0.23	150.6
0.001	0.11	70.0
0.0005	0.08	31.2
0.0001	0.07	27.3

For the differently loaded samples, the photocatalytic H_2_ production activities were measured in a quartz cell reactor illuminated with a 365 nm light‐emitting diode (LED) at 65 mW cm^−2^ intensity (more details are given in the experimental section). The results of the photocatalytic experiments in Figure 2e show that for all Pt SA deposited samples, a linearly increasing H_2_ volume is obtained over illumination time (i.e., H_2_ is evolving with a constant rate on all samples). The rates in Table 1 are determined from the slopes of these curves in Figure 2e–they vary from ≈27 to ≈150 µL h^−1^ depending on the Pt surface loading (see Figure 2f). Note that all the photocatalytic hydrogen production rates are significantly higher than for the non‐Pt loaded sample (≈3.4 µL h^−1^). From Figure 2f, it is apparent that the H_2_ production rates increase with Pt loading until saturation in performance occurs at 0.23 at.% Pt. At this point, an optimal Pt loading is reached, where a maximal H_2_ production efficiency is established with a minimal Pt amount. In other words, for Pt loadings (> 0.23 at.%), the amount of anchored Pt SAs on the titania surface is no longer rate‐relevant.^[^ ^51^ ^]^ This may be due to two effects: i) either a supercritical loading of equal reaction sites (enough co‐catalytic sites are provided to deal with all arriving photo‐generated electrons), or ii) the saturation of hot spots (i.e., all available highly reactive surface sites are fully SA‐occupied at a concentration of 0.23 at.%)—the latter implies that Pt SAs deposited at higher precursor concentrations are much less reactive. For either hypothesis (i or ii), there are arguments in the literature.^[^ ^49^, ^51^, ^55^, ^57^ ^]^ It is noteworthy that from HAADF‐STEM, even at the highest Pt precursor solution concentration of 2 mm, the surface remains primarily decorated with Pt SAs (Figure S4, Supporting Information). Figure S5a,b (Supporting Information) shows statistical analysis of HAADF‐STEM images regarding Pt SA loading on the titania surfaces. The quantification was performed over multiple representative regions across the sample surface, and the resulting densities include an ± 10% variation, as indicated by error bars. For two different precursor concentrations, 0.005 and 2 mm, the total Pt SA densities are 4.1 × 10^5^ and 8.8 × 10^5^ µm^−2^, respectively, and this is well in line with the XPS data in Table 1 with Pt concentrations of 0.23 and 0.49 at.%, respectively (more detailed discussion is in Supporting Information). Although at higher Pt concentration some mild agglomerations to rafts occur, it is noteworthy that i) this is still not visible in the FE‐SEM images, and ii) the presence of the agglomerates does not contribute at all to the final reactivity of the surface. These findings are in line with previous conclusions that anatase surfaces, when decorated with ≈10^5^–10^6^ Pt SAs µm^−2^, show a maximized photocatalytic activity under typical illumination conditions.^[^ ^48^, ^49^, ^51^ ^]^ Importantly, long‐term stability tests (Figure S1d, Supporting Information) confirm that the hydrogen evolution performance remains stable for continuous illumination, despite partial aggregation of Pt single atoms into nanoparticles (as observed in Figure S1b, Supporting Information). This observation supports that the remaining Pt SAs maintain catalytic activity and that the formed nanoparticles are not the primary active species. Additionally, no Pt leaching was observed by XPS (Figure S1c, Supporting Information), underlining the chemical robustness of the reactive‐deposited Pt SAs during photocatalysis.^[^ ^49^, ^51^, ^55^, ^57^ ^]^ A schematic representation of this photocatalytic process and proton reduction at Pt single atoms is illustrated in Figure S12 (Supporting Information).

**Figure 3:**
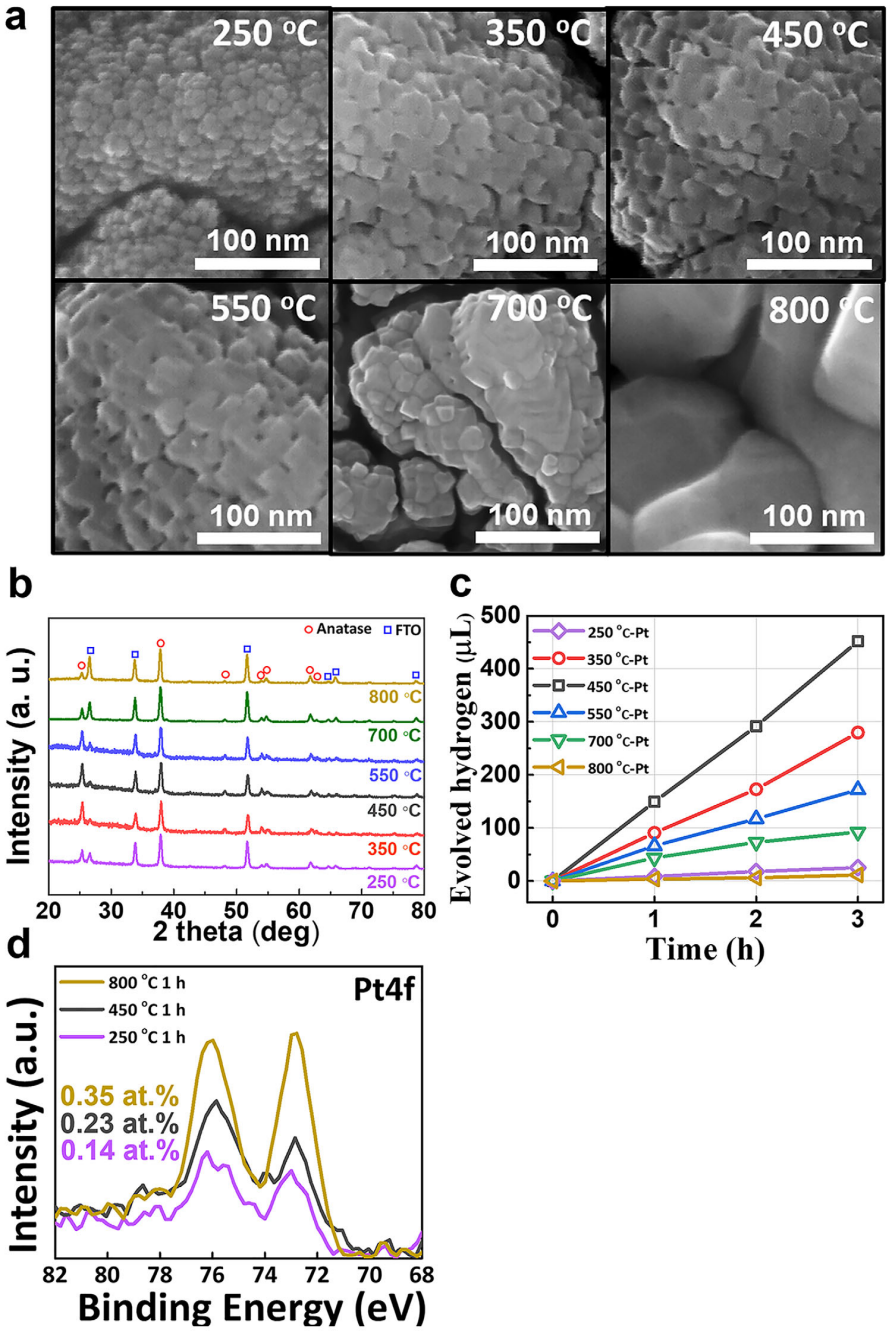
a) SEM images of 200‐nm TiO_2_ layers on FTO annealed at different temperatures, b) their XRD patterns, c) photocatalytic H_2_ evolution of the TiO_2_ layers annealed at different temperatures with a 0.005 mm Pt precursor decoration and d) their XPS spectra of Pt4f.

In the next step, we investigated factors of the substrate, i.e., the sputter‐deposited TiO_2_ layers, on the H_2_ production activity. First, we examined the influence of the annealing temperature in the range of 250–800 °C. **Figure** **3**a shows SEM images of sputtered 200‐nm‐thick layers after air annealing at various temperatures. The images reveal that annealing affects the grain size and at 800 °C, strong recrystallization occurs. This is in line with X‐ray diffraction (XRD) spectra (Figure 3b) that show, for all annealing temperatures from 250 to 800 °C, clear peaks of anatase (and tin oxide from FTO); no other polymorphs of TiO_2_ can be detected.^[^ ^54^ ^]^ From the Scherrer approach,^[^ ^60^, ^61^ ^]^ the average crystallite size grows from 18.9 nm (250 °C) to 22.3 nm (800 °C). In particular, the main anatase (101) peak at 2θ = 25.3° (JCPDS #21‐1272) increases in intensity up to 550 °C, indicating improved crystallinity. However, at higher annealing temperatures (≥ 700 °C), the anatase peaks decrease relative to the SnO_2_ (110) peak at 2θ = 26.6° (JCPDS #41‐1445) from the underlying FTO substrate.^[^ ^54^ ^]^ This trend suggests Sn diffusion into the TiO_2_ film, which can subtly influence the lattice structure and alter the apparent distribution of crystallographic planes. These differently annealed layers were then decorated with Pt SAs using the above optimized Pt precursor concentration (0.005 mm). The resulting photocatalytic H_2_ production rates are shown in Figure 3c. With increasing annealing temperature, the H_2_ production rate increases, until a maximum activity is reached for samples annealed at 450 °C (150.6 µL h^−1^). Annealing at higher temperatures leads to decreasing activity, and samples annealed at 800 °C have almost entirely lost their photocatalytic activity (3.9 µL h^−1^). This loss in activity can be attributed to significant diffusion of Sn from the FTO substrate into the TiO_2_ layer, resulting in a detrimental effect.^[^ ^54^ ^]^ Interestingly, XPS analysis (Figure 3d) shows a mild increase in Pt single‐atom loading for the titania surfaces annealed at higher temperatures. This may be due to an increase in the number of docking sites (due to the changes in the crystallographic features), or to an increase in the effective surface area of the TiO_2_ layer as a result of grain growth and morphological changes induced by high‐temperature annealing. The larger grains may lead to a more pronounced surface roughness, thereby increasing the actual surface area available for Pt adsorption. Despite the increased Pt loading, this additional amount of co‐catalyst is not photocatalytically favorable. This indicates that the decrease in photocatalytic activity at higher annealing temperatures is primarily due to degradation of the TiO_2_ electronic properties—such as increased recombination from Sn diffusion—rather than any significant change in the Pt SA distribution. This trend further supports that Pt SA density is not the dominant factor in determining catalytic efficiency. The hydrogen evolution rate reaches a maximum at 450 °C, even though Pt loading is slightly higher at 800 °C. Instead, the results strongly suggest that the electronic quality of the TiO_2_ film—especially its conductivity and defect properties—is the primary parameter controlling performance. Key are the inferior electronic properties of the layer annealed at 250 °C (not sufficiently crystallized to anatase) and the Sn uptake at T > 450 °C (providing recombination centers in the anatase^[^ ^62^ ^]^). This deterioration in the electronic properties of the sputtered TiO_2_ layer on FTO is visible in solid‐state current‐voltage (*I*–*V)0* measurements of the differently annealed layers (Figure S6, Supporting Information). The *I*–*V* curves reveal that annealing at 450 °C achieves a maximized conductivity, i.e., represents a balance between beneficial crystallinity of TiO_2_ film and minimal Sn diffusion from the FTO substrate. At 250 °C, the insufficient anatase crystallization leads to a higher defect density, thereby compromising charge transport.^[^ ^54^, ^63^ ^]^ Conversely, at 800 °C, excessive Sn incorporation introduces electronic defect states that hinder electron mobility.^[^ ^64^ ^]^ Consequently, the TiO_2_ layer annealed at 450 °C exhibits superior electronic properties, as demonstrated by enhanced vertical (Figure S6a, Supporting Information) and lateral (Figure S6b, Supporting Information) charge transport, which is well in line with the photocatalytic activities in Figure 3c.

**Figure 4:**
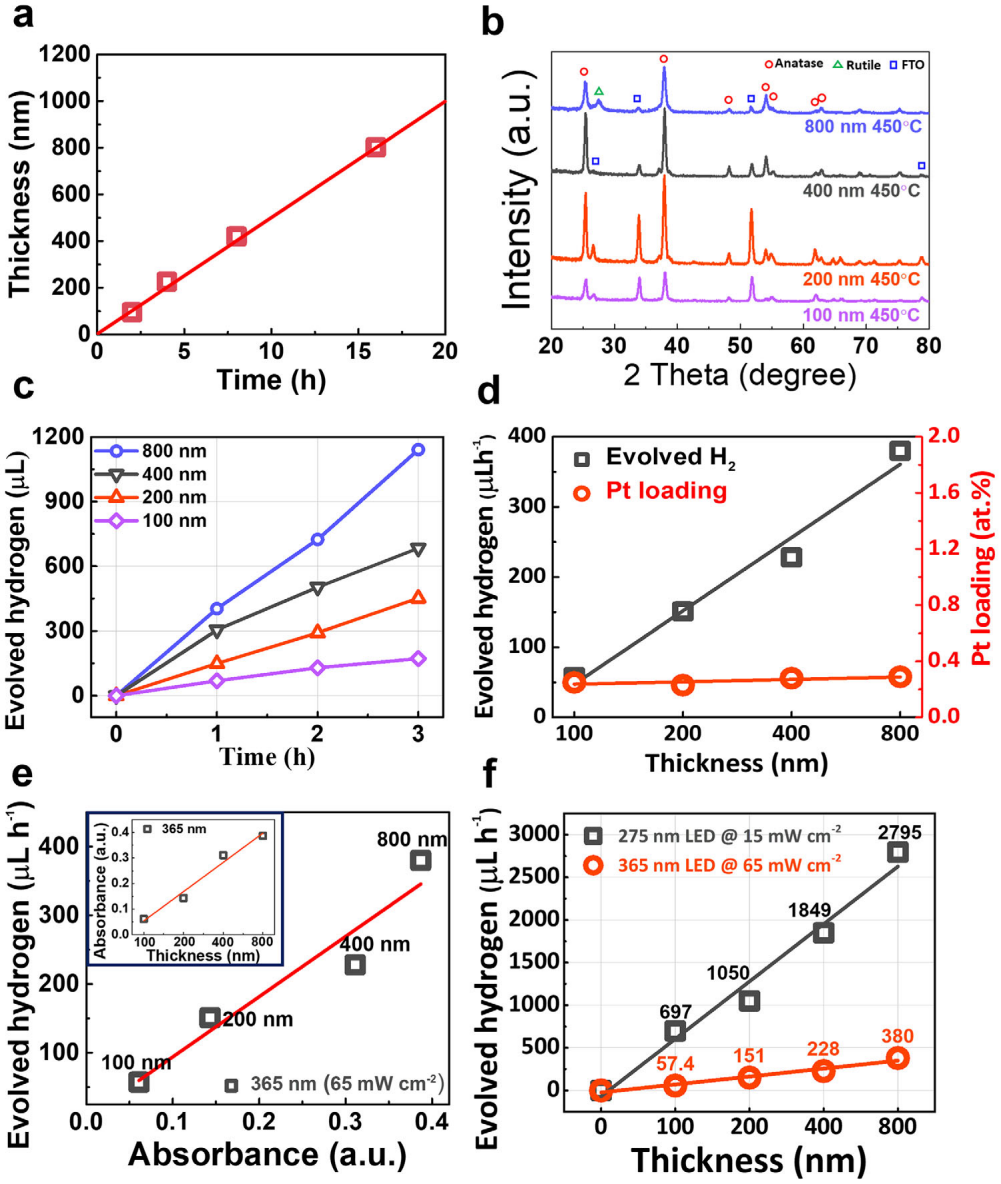
a) TiO_2_ film growth as a function of deposition time, b) XRD patterns for TiO_2_ films with different thickness after air‐annealing at 450 °C, c) their H_2_ production over time decorated with 0.005 mm of Pt precursor (at 365 nm, 65 mW cm^−2^), d) their H_2_ production rates (black) and Pt surface loading (red) on the titania surface as a function of thickness, e) H_2_ production rates with different TiO_2_ thickness as a function of absorbance spectra (inset: absorbance spectra over thickness), and f) H_2_ production rate comparison in different illumination wavelengths (275 nm vs. 365 nm) as a function of titania thickness.

In the following, we investigated titania layers of different thicknesses, varying from 100 to 800 nm, produced by a variation of the sputter‐deposition time from 2 to 16 h. The graph in **Figure** **4**a shows linear film growth over deposition time and thus a constant growth rate (51.6 ± 4.6 nm h^−1^) over time. Cross‐sectional SEM images of the resulting layers are shown in Figure S7 (Supporting Information). After deposition, all layers underwent annealing in air at the optimized temperature of 450 °C. For all layers, XRD patterns (Figure 4b) reveal prominent anatase peaks, but for the thickest titania layer (i.e., 800 nm), minor rutile contributions, primarily at 2θ = 27.4°, can be detected (JCPDS #21‐1276). For layers with varying thicknesses, photocurrent spectra were acquired. As seen in Figure S8 (Supporting Information), the incident photon‐to‐current efficiency (IPCE) results show different peak positions for the maximum photocurrent. In thinner layers (100 and 200 nm), the maximum IPCE is observed at ≈300 nm of wavelength, whereas in thicker films (400 and 800 nm), the peak shifts toward longer wavelengths (320 and 335 nm, respectively). This shift can be attributed to the increased optical path length in the thicker films, which allows photons with longer wavelengths to be absorbed more effectively. After the layers were Pt single‐atom decorated (0.005 mm H_2_PtCl_6_), they were examined for their photocatalytic hydrogen production efficiency (Figure 4c). Evidently, as the layer thickness increases, the total H_2_ production rises linearly from 172.2 µL (100 nm) to 1140.7 µL (800 nm) over 3 h, suggesting that the enhanced photocatalytic H_2_ production rates with increasing thickness (Figure 4d, black) are due to enhanced light absorption.

To ensure that the observed increase in hydrogen evolution is not due to variations in co‐catalyst (Pt SA) loading, XPS measurements were conducted to assess the Pt SA surface coverage (Figure 4d, red). The results confirm a constant Pt surface loading of ≈0.26 ± 0.03 at.% across all film thicknesses (see also Table S1 and Figure S9, Supporting Information). Therefore, the linear increase in photocatalytic activity with film thickness is attributed to enhanced light absorption and overall electron generation in thicker films, rather than to an increase in Pt active sites. Importantly, even for the thickest TiO_2_ film (800 nm), this Pt SA loading is sufficient to utilize the full flux of photogenerated electrons, confirming that the optimal Pt density is effective even under high charge carrier generation conditions. Light absorption spectra (Figure S10a, Supporting Information) confirm a linear increase of the absorbance at 365 nm (the excitation wavelength) with thickness (Figure 4e inset), and the linear correlation between the corresponding H_2_ generation rate and the layer thickness (Figure 4e) clearly shows that the enhanced light absorption in thicker TiO_2_ films determines the overall photocatalytic performance. To further examine this correlation, we performed measurements using a shorter excitation wavelength (275 nm) that is much strongly absorbed than the 365 nm (for more details see discussion to Figure S10, Supporting Information). Figure S11 (Supporting Information) presents photocatalytic hydrogen evolution measurements under 275 nm illumination (at 15 mW cm^−2^) as a function of thickness, which also reveals a linear dependence of the hydrogen evolution rate (R_H_) on film thickness. Notably, Figure 4f shows that the overall R_H_ efficiency at 275 nm is significantly higher than at 365 nm, roughly corresponding to the considerably greater absorption (A) at 275 nm (considering absorption A_275_ ≈ 23A_365_ and illumination power (P) with P_365_ ≈ 4P_275_).

Most importantly, our findings clearly establish that a Pt surface decoration with a SA density of ≈4.1 × 10^5^ µm^−2^ corresponding to a XPS loading of 0.26 ± 0.03 at.% Pt is fully sufficient to manage all electron flux conditions in this work, including higher fluxes established by thicker layers and under high‐energy (short‐wavelength) illumination. These results strongly indicate that for such loading (4 × 10^5^ µm^−2^), the photocatalytic hydrogen evolution kinetics are primarily controlled by the total amount of light absorbed within the TiO_2_ photoabsorber (not by the Pt co‐catalyst). Therefore, our study underscores the concept of a critical co‐catalyst density—a minimal threshold of catalytically active, well‐dispersed single atoms needed to ensure full utilization of photogenerated charge carriers. Once this threshold is met, additional SA loading no longer enhances photocatalytic activity and may indeed lead to suboptimal configurations, such as Pt rafts or clusters, that fail to contribute proportionally to enhanced reaction kinetics. For the flat anatase layers studied here, this critical density was determined at ≈4 × 10^5^ µm^−2^, achievable by simply exposing the surface to a dilute (5 µm) H_2_PtCl_6_ solution.

An additional important advantage of our flat titania layers produced by sputter deposition lies in the natural placement of Pt SAs (i.e., only at the semiconductor surface perpendicular to the shortest electron transport path—i.e. where electron transfer reactions actually take place. In contrast to classical powder‐based systems, where Pt SAs are randomly distributed throughout the 3D structure, our surface‐confined decoration ensures that all catalytic centers are effectively positioned exactly where they are needed. This targeted surface‐only deposition not only maximizes catalytic efficiency per Pt atom but also drastically reduces the required total Pt loading. Consequently, this methodology not only is an ideal geometry for parameter studies in photocatalysis (and photoelectrochemistry) but it also is a catalyst/absorber arrangement that allows for optimizing the usage of precious metals without compromising catalytic performance.

## 3 Conclusion

In this work, we systematically investigate photocatalytic hydrogen generation of flat, DC‐sputtered, anatase TiO_2_ thin films decorated with Pt SAs by reactive deposition, and identify the fundamental factors governing SA attachment, activity, and photocatalytic performance. First, we address key parameters regarding Pt loading and show that reactive deposition follows Langmuir‐type behavior, indicating that deposition occurs at a finite number of surface sites. We find that a critical Pt SA density of ≈4 × 10^5^ µm^−2^ (≈0.26 at.%) is sufficient to achieve the maximum hydrogen evolution rate, even under intensified excitation (e.g., short‐wavelength ultraviolet (UV) illumination) and increased absorber thickness.

Beyond this threshold, additional Pt loading provides no benefit and can even reduce efficiency due to clustering or inactive site formation. This clearly establishes that the key performance‐limiting factor is not the density of co‐catalytic sites, but rather the absorber's capacity for light harvesting and charge carrier transport. The strategic, surface‐only placement of Pt single atoms ensures they are positioned directly at the photocatalytically active interface, where photogenerated electrons are transferred. Compared to conventional powder‐based systems with randomly distributed Pt atoms, our thin‐film configuration maximizes atom efficiency, enabling high catalytic performance with minimal noble metal consumption. The concept of a “critical single‐atom density” established here—closely tied to absorber properties and light excitation conditions—defines a threshold beyond which additional Pt contributes little to performance. These findings provide design principles for SAC‐based photocatalysts that integrate precise atomic placement with optimized absorber architectures and resource efficiency.

## 4 Experimental Section

### Substrate Preparation for Reactive Magnetron Sputtering

The preparation of the TiO_2_ film has been described in previous work.^[^ ^54^ ^]^ In short, titania layers were deposited on FTO or silicon dioxide wafers (SiO_2_) by DC‐MS in a reactive mode, using O_2_ as the reactive gas. The FTO glass (7 Ω sq^−1^, 1.5 cm × 2.5 cm, Solaronix) was sequentially sonicated in acetone, ethanol, and deionized (DI) water for 15 min each, and then dried with a nitrogen stream. For the SiO_2_ wafer substrate (MicroChemicals), the wafer was cleaned for 5 min using an O_2_ plasma cleaner (SmartPlasma, Plasmatechnology). Afterward, the substrates were inserted into the sputter load‐lock, where the vacuum was equalized to that of the main sputter chamber (SC, Createc – SP‐P‐US‐6M‐3Z). The cleaned substrates in the load‐lock were then transferred to a sample holder in the sputter chamber for the subsequent sputtering processes.

### Ti Target Conditioning

Before depositing the compact oxide layer, a titanium target (99.995%, dia. 127 mm × 8 mm, HMW–Hauner GmbH & Co. KG) was conditioned under an Ar atmosphere to eliminate the passivating oxide layer formed during previous sputtering. Initially, the base pressure (P_b_) was adjusted to ≈1.0 × 10^−7^ mbar. For the target conditioning, the pump speed of the sputter chamber was first reduced to 20%, and then argon gas (4 sccm) was introduced into the sputter chamber. When the working pressure (P_w_) stabilized at ≈1.4 × 10^−3^ mbar, 50 W of DC power was initially applied, increasing gradually up to 500 W and kept for 10 min (shutter kept closed).

### Fabrication of TiO_2_/Ti/FTO Samples

A thin layer of Ti metal was deposited onto FTO prior to the TiO_2_ layer deposition. The substrate holder was set to rotate at 50 rpm for uniform deposition. Subsequently, 150 W of DC power was applied for 10 s to deposit the Ti interlayer on FTO glass without substrate heating. After that, the main TiO_2_ layers were deposited by DC‐MS in a reactive mode. Argon gas (10 sccm) and oxygen gas (5 sccm) were introduced into the sputter chamber until P_w_ stabilized at ≈6.7 × 10^−3^ mbar. The oxygen fraction (*f*_O2_) was fixed at 0.33, and its partial pressure (P_O2_) was ≈2.2 × 10^−3^ mbar. Mass flow controllers (MFC, MKS Instruments, Inc.) precisely controlled the flow rate and partial pressure of the gases. The distance between the titanium target and the FTO substrates was maintained at 115 mm. Sputter deposition was carried out at room temperature under a circulating cooling water system set at 18 °C. The sputtering process was performed at 500 W for different deposition times depending on the desired thickness (100–800 nm). To ensure uniform film thickness, the substrate holder was continuously rotated at 50 rpm during deposition. The deposited layers were then annealed at different temperatures for 1 h in an air tubular furnace.

### Pt Single Atom Deposition on TiO_2_/Ti/FTO

To carry out the Pt single atom deposition, deionized water (10 mL, 18.2 MΩ) containing diluted H_2_PtCl_6_ (Metakem) precursor at different concentrations was prepared in quartz cells, followed by Ar purging. After 15 min purging with Ar to remove residual gases, the annealed titania samples were immersed in the quartz cell. Then, the cell was sealed and kept in the dark for 1 h. The samples were then sequentially immersed in ethanol and deionized water for 15 min each, and finally dried in a nitrogen stream.

### H_2_ Evolution Reaction

For the photocatalytic hydrogen evolution tests, a quartz reactor (17 mL) containing 10 mL 50 vol.% aqueous methanol solution was used. Methanol acts as a hole scavenger, enabling fast consumption of photogenerated holes and thus reducing charge carrier recombination. In addition, since the oxygen evolution reaction is typically the rate‐determining step in overall water splitting and is inhibited by the presence of dissolved oxygen, methanol was used along with Ar purging to isolate and stabilize the hydrogen evolution half‐reaction.^[^ ^39^, ^65^, ^66^ ^]^ In this study, photocatalytic activity was evaluated based on the irradiated geometric area of the thin films, which was fixed at 1 cm^2^. Given the planar thin‐film configuration, catalyst mass was not used as a normalization parameter. After Ar purging for 15 min, the reactor was sealed, and the samples were irradiated using two UV LEDs with different wavelengths and intensities. A 365 nm LED (D&S SPCM0800) and a 275 nm UV LED (D&S SP40×40275) was used with intensities of 65 and 15 mW cm^−2^, respectively. The samples were irradiated for 3 h, and the photocatalytically generated H_2_ volume was measured hourly using a gas chromatograph. The spectral full width at half maximum (FWHM) is ≈10–12 nm for the 365 nm LED and ≈13 nm for the 275 nm LED.

### Characterization

High‐resolution transmission electron microscopy (HRTEM) and HAADF‐STEM were carried out by FEI Titan G2 60–300. The surface and cross‐section morphologies of samples were investigated by FE‐SEM (S‐4800, Hitachi). The composition and chemical state of samples were analyzed by XPS (PHI5600). The peak deconvolution was carried out by MultiPak software, and all XPS spectra were aligned by a binding energy of Ti 2p (458.5 eV). The crystalline structure of the samples was determined by XRD (X'pert Philips MPD with a Panalytical X'celerator detector) by means of graphite monochromatized Cu Kα radiation (wavelength 1.5406 Å). The size of the TiO_2_ crystallites was estimated using the Scherrer equation.^[^ ^55^, ^56^ ^]^ The photocatalytic H_2_ was determined by a gas chromatograph (GCMS‐QO2010SE, SHIMADZU) with a thermal conductivity detector (TCD). The electronic properties of the samples were analyzed by two‐point probe *I*–*V* measurements using a Keithley 4200 Source Meter (Kleindiek nanotechnik). Absorbance measurements were performed using a UV–vis spectrophotometer (AvaLight‐DH‐S‐BAL, Avantes) in the wavelength range of 200–600 nm.

## Conflict of Interest

The authors declare that there is no conflict of interest.

## Author Contributions

H.K. wrote the original draft, review & editing, did investigation, validation, and visualization. N.D. did review & editing, validation, and visualization. Y.W. did investigation and visualization. P.S. did conceptualization, supervision, project administration, funding acquisition, and wrote the review & editing.

## Supporting information



Supporting Information

## Data Availability

The data that support the findings of this study are available from the corresponding author upon reasonable request.
