# Supplementary material for: Pt Single Atom Co‐Catalysts on Thin, Defined Anatase Layers: Critical Factors for Photocatalytic Hydrogen Generation
Source: Small. 2025 Sep 12;21(43):e07662. doi: 10.1002/smll.202507662 (PMC12571210; doi:10.1002/smll.202507662)
Supplement: Supplementary file 1 — Supporting Information [file SMLL-21-e07662-s001.docx]

**Supporting Information**

**Pt Single Atom Co-Catalysts on Thin, Defined Anatase Layers:**

**Critical Factors for Photocatalytic Hydrogen Generation**

*Hyesung Kim^1^, Nikita Denisov^1^, Yue Wang^1^, and Patrik Schmuki^1,2^**

^1^Department of Materials Science WW4-LKO, Friedrich-Alexander-University of Erlangen-Nuremberg, Martensstrasse 7, 91058 Erlangen, Germany

^2^Regional Centre of Advanced Technologies and Materials, Šlechtitelů 27, 78371 Olomouc, Czech Republic

*Author to whom correspondence should be addressed:

E-mail: schmuki@ww.uni-erlangen.de

**Table S1.** Pt loading (by XPS) on TiO_2_ surface for layers of different thickness deposited by 0.005 mM of precursor solution concentration

| Titania thickness (nm) | Pt loading (at. %) | Residual Cl (at. %) |
| --- | --- | --- |
| 100 | 0.25 | - |
| 200 | 0.23 | - |
| 400 | 0.28 | - |
| 800 | 0.29 | - |


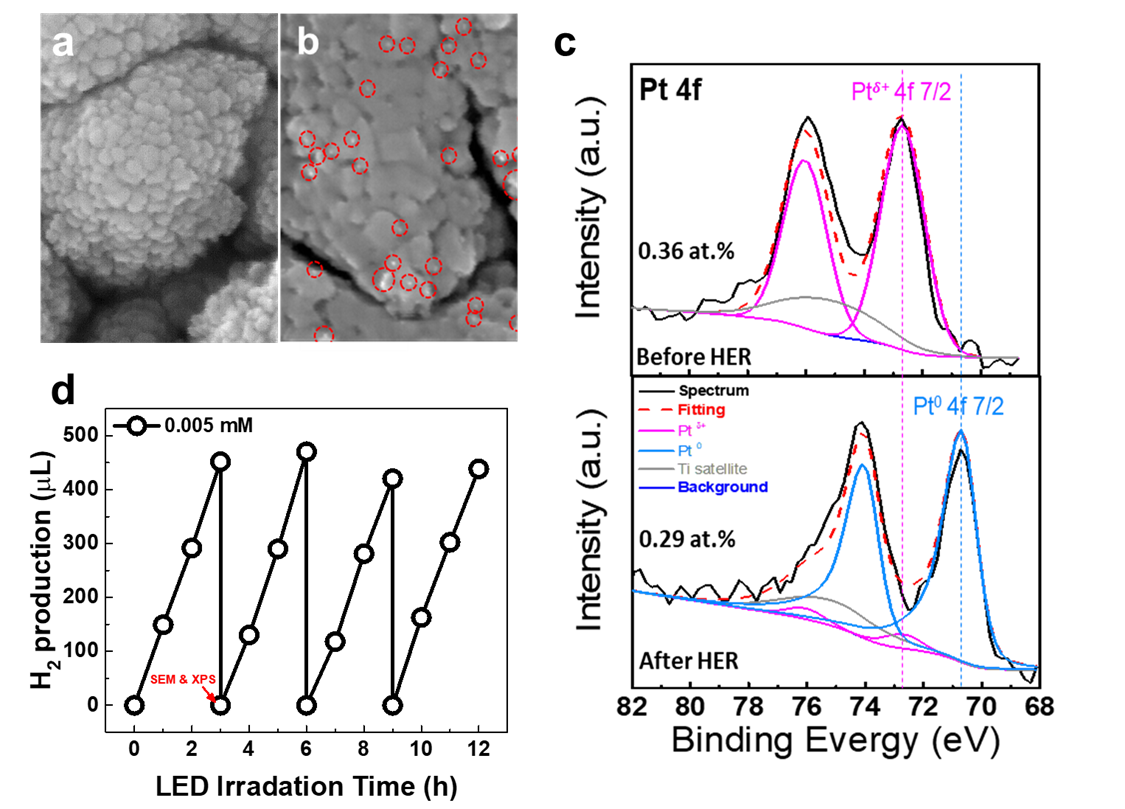


**Figure S1.** a) SEM image of a 200 nm-thick sputtered TiO_2_ film on FTO decorated with Pt single atoms before photocatalytic HER, b) SEM image of the same sample after 3 h of photocatalytic H_2_ evolution, agglomerated Pt nanoparticles are highlighted with red circles, c) XPS Pt 4f spectra before and after illumination, and d) H_2_ evolution stability test for 12 h (LED: 365 nm, 65 mW cm^-2^).


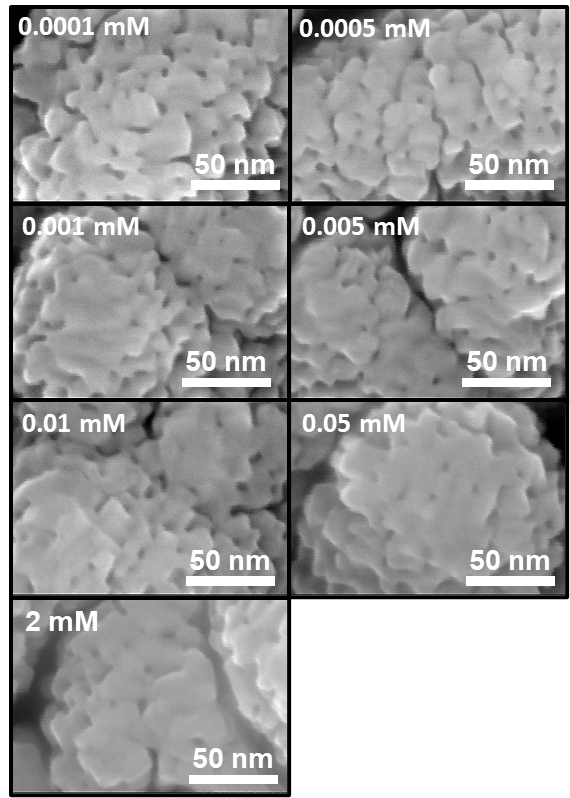


**Figure S2.** SEM images of Pt SA decorated 200-nm-thick TiO_2_ layers on FTO. Pt precursor (H_2_PtCl_6_) concentrations are from 0.0001 mM to 2 mM, showing no agglomerated Pt nanoparticles.

**Figure S3**. XPS spectra of residual Cl 2p peaks on titania surface decorated in different Pt precursor concentrations.


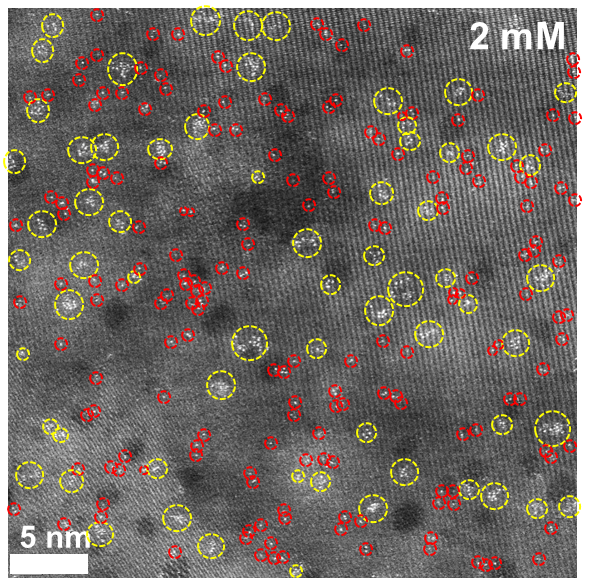


**Figure S4.** HAADF-STEM image of Pt decorated 7 nm TiO_2_ on TEM grid with a Pt precursor solution concentration of 2mM. SAs, dimers and trimers are marked with red circles, and 2D rafts are marked with yellow circles.

**Pt loading density statistics:** At a precursor concentration of 0.005 mM (Fig. S5a), Pt predominantly exists as isolated single atoms (SAs). Among the total Pt density of 4.1 × 10^5^ μm^-2^, approximately 81.7% (3.4 × 10^5^ μm^-2^) are isolated SAs. The remaining species consist of dimers (4.3 × 10^4^ μm^-2^; ~ 10.5%), trimers (2.7 × 10^4^ μm^-2^; ~ 6.6%), and a minimal number of 2D rafts (>0.3 nm), with a density of 5.0 × 10^3^ μm^-2^ (~ 1.2%). In contrast, at a higher precursor concentration of 2 mM (Fig. S5b), the total Pt density increases to 8.8 × 10^5^ μm^-2^, but the density and fraction of isolated SAs decrease significantly to 1.2 × 10^5^ μm^-2^ (~ 13.6%). The majority of Pt species at this loading are present as 2D rafts (6.5 × 10^5^ μm^-2^; ~ 73.9%), while the densities of dimers (7.4 × 10^4^ μm^-2^) and trimers (3.6 × 10^4^ μm^-2^) remain on the same order of magnitude as those observed at 0.005 mM. The bar charts represent mean values extracted from multiple HAADF-STEM images, and the error bars indicate an approximate ± 10% variation due to local surface inhomogeneities.


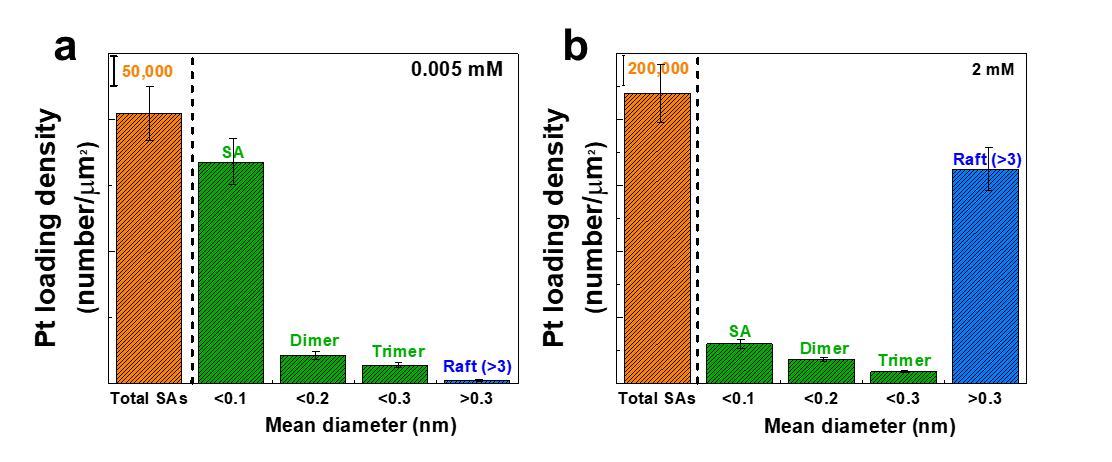


**Figure S5.** Statistical evaluation of Pt species on TiO_2_ surfaces for precursor concentrations of a) 0.005 mM and b) 2 mM H_2_PtCl_6_. Bar charts show the areal densities of isolated SAs, dimers, trimers, and 2D rafts, as well as the corresponding mean particle diameters. Error bars indicate approximately ± 10% variation across multiple HAADF-STEM images.

**I-V measurements:** One measurement (Fig. S6a) is conducted between a Pt contact and the FTO substrate, and the other (Fig. S6b) is between two Pt contacts on the TiO_2_ layer with a gap distance of 1.5 cm. These two configurations were used to provide a comprehensive understanding of the electronic behavior of TiO_2_ layer by screening the applied voltage from -1 V to +1 V. The first configuration evaluates the vertical charge transport through the TiO_2_ layer, which is crucial for applications involving current flow perpendicular to the substrate back contact (FTO). The second configuration focuses on the lateral charge transport within the TiO_2_ layer, providing insight into its intrinsic electronic properties independent of the FTO substrate. The measurements were performed after annealing the TiO_2_ layers at 250 °C, 450 °C, and 800 °C.


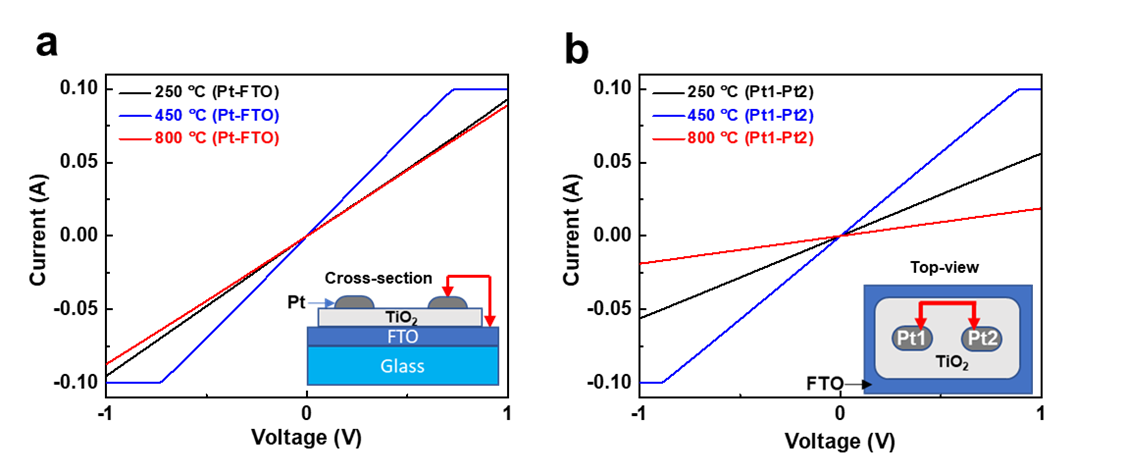


**Figure S6.** Solid state I-V measurements for 200-nm-think sputtered TiO_2_ layers on FTO air-annealed in different temperatures (250 °C to 800 °C). a) applied voltage through Pt metal contact to FTO, and b) through Pt metal contact 1 (Pt 1) to Pt metal contact 2 (Pt 2).


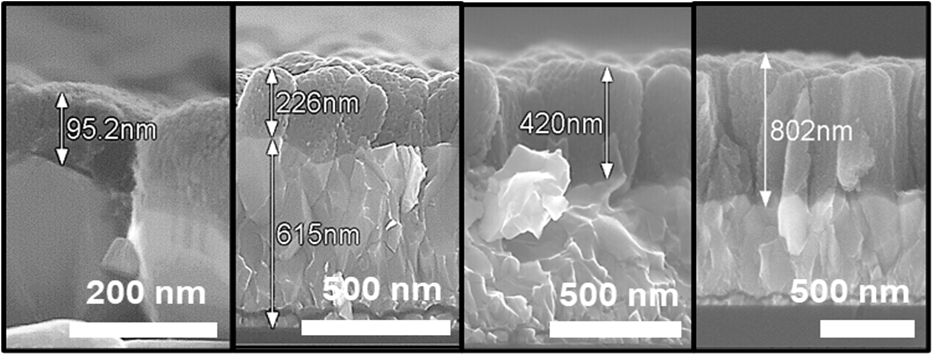


**Figure S7**. SEM cross-sections of titania layers in different thickness on FTO (sputtering deposition times are from the left 2 h, 4 h, 8 h and 16 h).


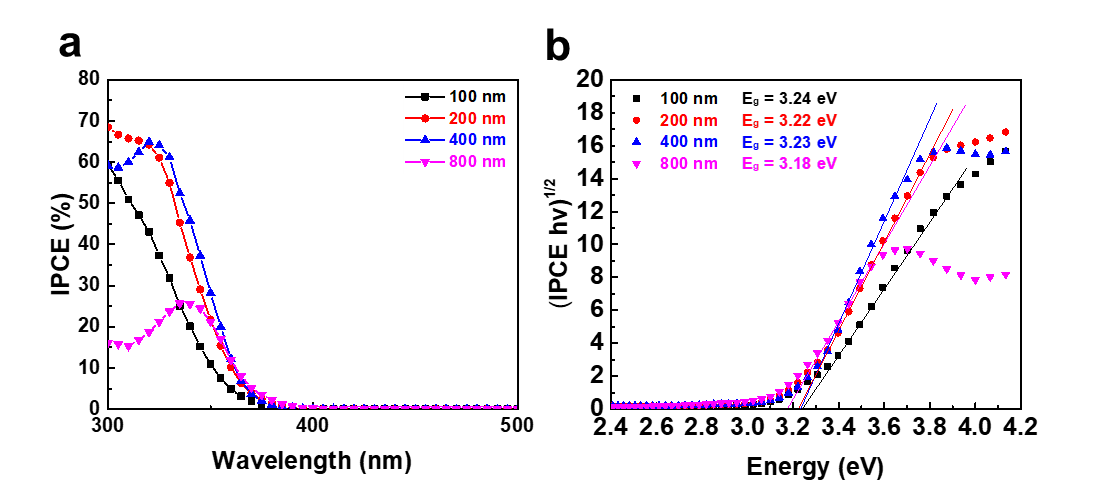


**Figure S8**. IPCE spectra of TiO_2_ layers on FTO in different thickness (100 – 800 nm) annealed at 450 ℃.

**Figure S9.** XPS spectra of Pt decorated (0.005 mM precursor concentration) on TiO_2_ / FTO (annealed at 450 °C) for different TiO_2_ layer thickness (100 nm – 800 nm).

**Absorbance spectra:** Absorption for TiO_2_ in different layer thickness at 275 nm are approx. 18 – 28 times higher than at 365 nm, as shown in Fig S10b. In other words, the higher hydrogen production can be fully accounted 18 – 28 times (200 nm and 100 nm, respectively) by the increased absorption at shorter wavelength. It is also noteworthy that the increase in H_2_ production rates at 275 nm is about 7 – 12 times higher than that at 365 nm (Fig. 4f), due to the lower light intensity of 275 nm (15 mW cm^-2^) compared to 365 nm (65 mW cm^-2^).


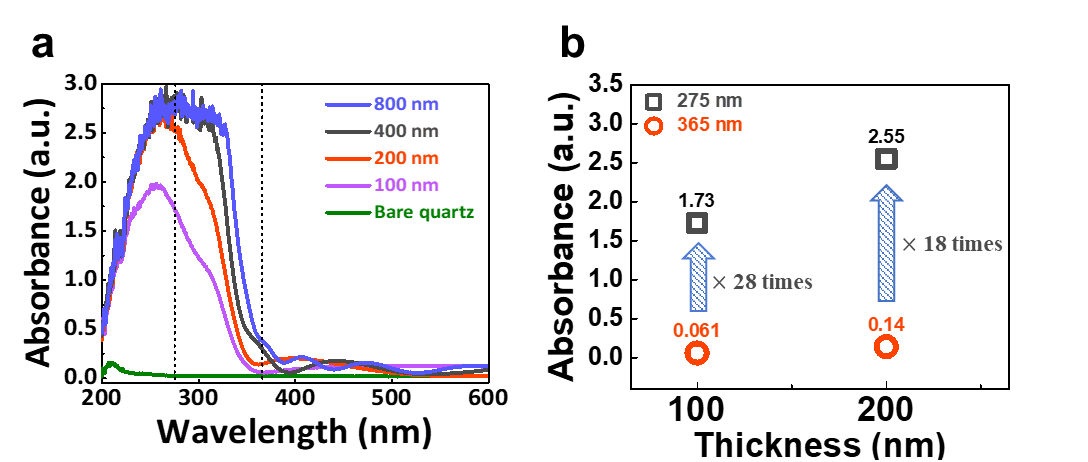


**Figure S10.** a) Absorbance spectra of the sputtered TiO_2_ layers in different thickness, and b) absorbance data points for the comparison in different TiO_2_ layers (100 nm and 200 nm) as a function of wavelength of light at 275 nm and 365 nm.

**Figure S11.** Photocatalytic H_2_ evolution of TiO_2_ layers with different thickness over 3-hour period (with Pt: 0.005 mM and 275 nm LED with 15 mW cm^-2^).


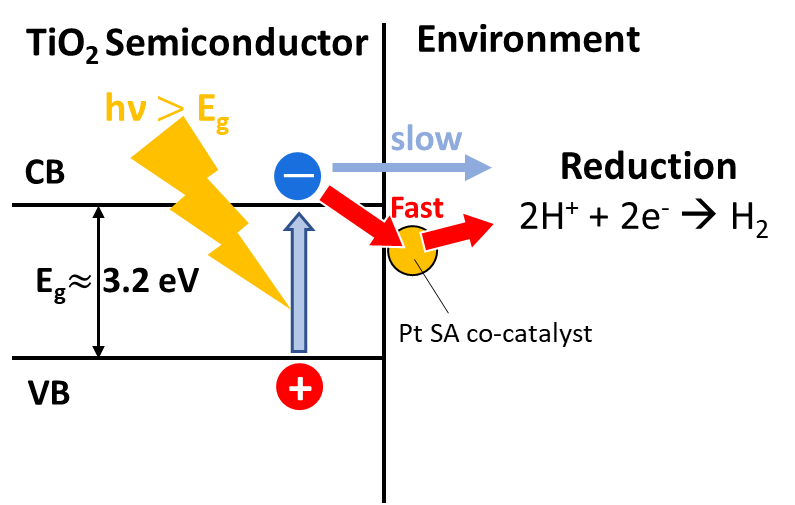


**Figure S12**. Schematic illustration of the photocatalytic H_2_ evolution mechanism on Pt single-atom-decorated TiO_2._ ^[a]^

Reference

[a] S. Wu, P. Schmuki, *Adv. Mater.* **2025**, *37*, 2414889.
